# Dietary phytochemical and metabolic disease prevention: Focus on plant proteins

**DOI:** 10.3389/fnut.2023.1089487

**Published:** 2023-01-25

**Authors:** Song-hong Yang, Gabriel Tao, Liu Yang, Xiaohui Wu, Jing-wen Liu, Fatima Dagher, Shi-yi Ou, Yuan Song, Jun-qing Huang

**Affiliations:** ^1^School of Pharmaceutical Sciences, Taizhou University, Taizhou, China; ^2^Department of Pharmacological and Pharmaceutical Sciences, College of Pharmacy, University of Houston, Houston, TX, United States; ^3^Department of Food Science and Engineering, Jinan University, Guangzhou, China; ^4^Guangzhou Key Laboratory of Formula-Pattern Research Center, School of Traditional Chinese Medicine, Jinan University, Guangzhou, China; ^5^The First Affiliated Hospital, Jinan University, Guangzhou, China

**Keywords:** food waste, inflammation, oxidative stress, circadian rhythm, metabolic disease

## Abstract

Plant-based functional foods have attracted increasing research interest to validate their use in preventing metabolic disease. Since it is increasingly recognized that inflammation, oxidative stress, and circadian rhythm play vital roles in various metabolic diseases, including diabetes, obesity and non-alcoholic liver disease, plant proteins, protein hydrolysates, and food extracts that intervene in these biological processes are promising dietary supplements to prevent metabolic diseases. Here, we reviewed the recent research on plant-based foods used for metabolic disease prevention and provided new perspectives regarding the current study gaps and future directions in this field.

## 1. Introduction

Plant-based functional foods have been used to prevent metabolic disorders such as type 2 diabetes, obesity, and cardiovascular diseases for years (1–5). However, the active components of many of these compounds, as well as the mechanism underlying these beneficial effects, have not yet been clearly elucidated. It is well-known that inflammation and oxidative stress promote the development of many metabolic diseases (6, 7). In particular, it has been shown that proinflammatory cytokines have profound impacts on glucose metabolism, lipogenesis and xenobiotic metabolism in the liver, which makes inhibiting inflammation a potential strategy to prevent the incidence of diabetes and non-alcoholic fatty liver disease (NAFLD) (8, 9). Markova et al. (10) recently reported that a diet high in plant protein significantly reduced liver fat and improved insulin resistance and the inflammatory response in diabetes patients. Recently, the role of the circadian clock in the development of metabolic syndromes has been revealed (11). An increasing number of studies have reported that numerous plant-based functional foods alter the regulation of circadian clock-related gene expression, but many details remain unclear (12).

In this review, we summarized the recent studies on plant-based foods for metabolic disease prevention and provide new perspectives on the current study gaps and future directions in this field. We first discussed the inflammatory reaction, oxidative damage and circadian rhythm regulation in diabetes and obesity. Next, we reviewed plant-based foods with anti-inflammatory and antioxidant functions and those that affect circadian rhythm, with a focus on plant proteins and active extracts.

## 2. Role of inflammation, oxidative stress, and the circadian rhythm in metabolic diseases

**FIGURE 1:**
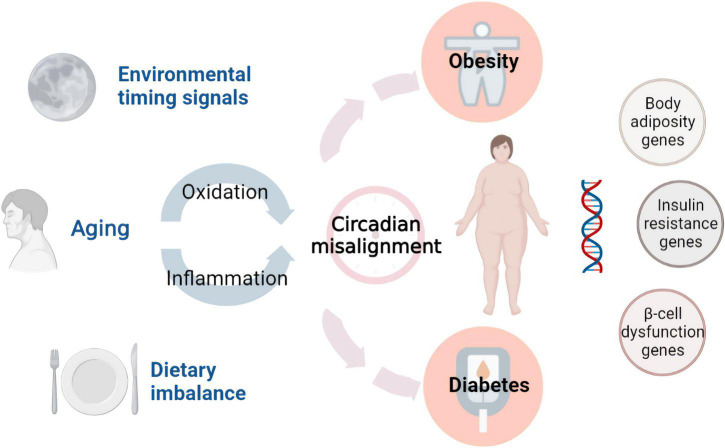
Environmental factors and endogenous damage in metabolic diseases. Functional foods prevent diseases by interfering with these factors.

Accumulating evidence suggests that proinflammatory pathways, redox signaling, and the circadian clock play important roles in the initiation and development of metabolic diseases (Figure 1). Many metabolic syndromes are associated with chronic inflammation, including tissue infiltration by monocytes, macrophages, and lymphocytes (13). The two major characteristics of type-2 diabetes (T2D) are insulin resistance and pancreatic β cell dysfunction (14–16). The finding that islet inflammation is involved in pancreatic β cell dysfunction highlights the importance of inflammation in these diseases. Recent evidence has indicated that intraislet macrophages are the main source of the increased proinflammatory cytokines in diabetes. Thus, targeting islet inflammation is an effective therapeutic strategy for T2D (17). Regarding the role of inflammation in obesity, most studies have focused on adipose tissue inflammation. When excessive accumulation of fat occurs in adipocytes, the inflammatory response can be detected in these cells. Gut-derived substances, dietary components or metabolites induce adipose tissue inflammation (18, 19). Alternatively, the rapid expansion of adipose tissue increases the inflammatory response through adipocyte death, hypoxia, and mechanotransduction arising from interactions between cells and the extracellular matrix (20). NAFLD causes liver dysfunction and is typically associated with advanced diabetes and obesity (21, 22). It is increasingly recognized that mediators released from immune cells and adipocytes impair insulin signaling in the liver (23). The disruption of insulin function leads to insulin resistance, which contributes to the development of NAFLD. The NF-κB/IKKβ pathway, JNK and PPARγ are involved in inflammatory accumulation and the development of NAFLD (24).

Similar to chronic inflammation, oxidative stress occurs in diabetic and obese individuals. Oxidative stress greatly contributes to the development of metabolic disease-induced cardiovascular complications (25). High levels of metabolic activity and reactive oxygen species (ROS) are inevitable byproducts of mitochondrial respiration, and pancreatic β cells express fewer antioxidant enzymes. This imbalance may render pancreatic β cells susceptible to ROS. When glucose clearance is impaired, the increase in glycolytic flux increases ROS production in pancreatic β cells. In addition to these effects, hyperglycemia and hyperinsulinemia promote the generation of hydrogen peroxide in β cells. ROS and hydrogen peroxide induce oxidative damage to ribonucleic acids, proteins, and lipids. Consequently, the function of pancreatic β cells can be damaged by ROS through multiple mechanisms, including decreased enzyme activity, dysregulated gene expression, and apoptosis (26). The production of ROS is promoted in adipose tissue during obesity and is accompanied by increased NADPH oxidase expression and the inhibition of antioxidative enzymes. In adipocytes, elevated levels of fatty acids increase oxidative stress and dysregulate adipocytokines, including adiponectin, plasminogen activator inhibitor-1, IL-6, and monocyte chemotactic protein-1. A recent study revealed that the redox state of adipose tissue is a therapeutic target for obesity (27).

In addition to inflammation and oxidative stress, the role of the circadian rhythm in metabolic diseases has been increasingly recognized in recent years (28, 29). In the liver, the circadian clock is be associated with metabolism and energy homeostasis. This is achieved by mediating the expression of metabolic enzymes and transporters. Glucose metabolism in healthy individuals is controlled by circadian rhythm and plays a key role in pancreatic β cell function. Exogenous melatonin significantly impairs metabolic homeostasis. This finding indicates that a higher risk of T2D is associated with circadian disruptions associated with shift work, social jet lag, late chronotype, and genetic risk (30). The core clock mechanism has been shown to regulate lipogenesis pathways. Recent molecular studies have shown the involvement of brain and muscle ARNT-like 1 (BMAL1) in the regulation of adipogenesis and lipid metabolism. The loss of BMAL1 leads to a significant decrease in adipogenesis and the expression of several key lipogenic factors (31).

## 3. Plant-based functional foods exert anti-inflammatory effects

As an essential immune response, inflammation is involved in the development of metabolic disorders. In recent years, plant proteins have received increasing attention due to evidence showing their potential to prevent metabolic disorders through the production of proinflammatory cytokines and reactive oxygen species (Table 1).

**TABLE 1:** Anti-inflammatory effects of plant-based functional foods.

Active components	Main source	Experimental model	Targets	Effects	References
Protein hydrolysates of brewers’ spent grain	Brewers’ spent grain	Rat splenocytes, macrophages, and T lymphocytes	TLR2, TLR4, NFκB, and MAPKs	Regulated the immune response *in vitro*	(32)
*Moringa* seed soluble fiber	*Moringa oleifera*	Murine splenocytes, RAW 264.7 macrophages	N/A	Increased the proliferation of splenocytes	(33)
Common bean hydrolysates	Common bean	LPS-induced RAW 264.7 macrophages	NF-κB pathway	Inhibited inflammation by modulating the NF-κB pathway	(34)
Methanolic extracts from leaves of *Capparis spinose*	*Capparis spinose*	Swiss mice	IFNγ, IL-17, and IL-4	Exerted an anti-inflammatory effect	(35)
		PBMCs	IL-17, IL-4	Promoted an anti-inflammatory response	(36)
Black raspberry	Black raspberry	Patients with metabolic syndrome	IL-6, TNF-α	Decreased inflammatory cytokine functions	(37)
SPE-Diol extract of extra virgin olive oil	Extra virgin olive oil	LPS/IFNγ-stimulated J774.A1 macrophages	NO, PGE2, TNFα, ILl-6	Anti-inflammatory effects	(38)

TLR, toll-like receptor; MAPK, mitogen-activated protein kinase; NF-κB, nuclear factor kappa-light-chain-enhancer of activated B cells; LPS, lipopolysaccharide; PBMCs, human peripheral blood mononuclear cells; IL, interleukin; TNF-α, tumor necrosis factor alpha; NO, nitric oxide; PGE2, prostaglandin.

### 3.1. Plant proteins that exert anti-inflammatory effects

Plant-based proteins have been the focus of research and are in extensive demand due to their bioactivity. Brewers’ spent grain (BSG) protein hydrolysates exert immunomodulatory effects *in vitro*, which can be beneficial in controlling inflammatory diseases (32). The regulation of the immune response includes Toll-like receptor 2 (TLR2), TLR4, NF-κB, and MAPKs. *Moringa oleifera* (moringa or drumstick) is a monogeneric plant. Its soluble seed fiber is a protease-resistant protein, which is a promising nutritional source to enhance the immune system. *Moringa oleifera* seeds significantly stimulated the proliferation of splenocytes and NO production (39). Common bean hydrolysates have been demonstrated to significantly inhibit the transactivation of NF-κB and the nuclear translocation of the NF-κB/p65 subunit, indicating that they can be used to treat inflammatory and oxidative diseases (34).

### 3.2. Crude extracts suppress inflammation

The methanolic extracts of leaves of *Capparis spinosa* showed anti-inflammatory effects *in vitro*. These extracts also inhibit membrane stabilization and exert anti-inflammatory effects in a mouse model (40). In addition, the extracts of *Capparis spinosa* leaves exhibited stronger anti-inflammatory activity than those of the fruits. An *in vivo* study in Swiss mice revealed that *Capparis spinosa* L. induced a significant decrease in immune cell infiltration, vasodilatation and dermis thickness by inhibiting the gene expression of cytokines, such as interferon gamma (IFN-γ), IL-17, and IL-4 (35). Another study reported that secondary metabolites of *Capparis Spinosa*, such as carotenoids and alkaloids, induced an anti-inflammatory response by suppressing IL-17 and inducing the IL-4 gene (41). This anti-inflammatory activity could be attributed to the presence of secondary metabolites in caper such as carotenoids, alkaloids (36, 42), and flavonoids or active protein hydrolysates. According to these studies, the *Capparis Spinosa* plant could be a valuable source of natural anti-inflammatory agents. Black raspberry has been reported to suppress superoxides, nitric oxide, and IL-6 (43). A randomized double-blind study reported that administering black raspberry for 12 weeks resulted in a significant decrease in inflammatory factors such as IL-6 and TNF-α (37). Another promising candidate for reducing the inflammatory response is extra virgin olive oil (EVOO). The anti-inflammatory activity of the SPE-Diol extract was assessed in response to LPS/IFNγ stimulation of J774.A1 macrophages (38). The results demonstrated the anti-inflammatory effects of the extract of EVOO, which decreased NO and PGE2 by inhibiting the expression of iNOS and COX-2. In addition, the anti-inflammatory effect was closely correlated with the total polyphenol content and DPPH scavenging activity. A recent study of *Citri Reticulatae Pericarpium* (CRP) showed that the beneficial effect of CRP extract on LPS-induced inflammation in RAW 264.7 cells involved inhibition of the MAPK and NF-κB signaling pathways. The extract acted on all three MAPK signaling pathways to reverse LPS-induced phosphorylation of JNK, ERK1/2, and p38 MAPK. Furthermore, the extract could inhibit the NF-κB signaling pathway by reducing the phosphorylation of NF-κB p65 (44).

## 4. Plant-based functional foods with antioxidant effects

Concerns about the long-term safety of synthetic antioxidants have led to increasing demand for natural antioxidants to lower the risk of oxidative stress-associated diseases. As the source of essential amino acids, plant proteins are considered functional ingredients that promote health. *In vitro*, the enzymatic hydrolysis of plant proteins to produce bioactive hydrolysates has been used to imitate protein degradation in the human gastrointestinal tract. The antioxidant properties of these hydrolysates have been reported in several studies (Table 2).

**TABLE 2:** Antioxidant effects of crude extracts of plant-based functional foods.

Active extracts	Main source	Experimental model	Beneficial effects	References
Sinapine; Sinapic acid	*Brassica napus* L. (Canola)	Human Caco2 cells Chinese hamster ovary cell lines	Reduces H_2_O_2_-induced cytotoxicity	(45)
Protocatechuic acid	*Clinacanthus nutans*	HFHC diet-fed rats	Increases serum antioxidant enzymes; Upregulates the expression of hepatic antioxidant genes	(46)
Anthocyanin	*Canarium odontophyllum*	Human liver cell lines	Cardioprotective effects	(47)
*P. emarginatus* oil extract	*Pterodon emarginatus*in	*Caenorhabditis elegans*	Protects against oxidative damage	(48)
*Citrus junos* extract	*Citrus juno*	3T3-L1 cells	Suppresses ROS production and lipid accumulation	(49)
Niazirin	*Moringa oleifera*	VSMCs from pigs–*in vitro* Diabetic mice-*in vivo*	Prevents diabetic atherosclerosis.	(50)

### 4.1. Plant proteins that exhibit antioxidant functions

The wide availability of plants belonging to the *Cucurbitaceae* family has prompted researchers to further explore the role and importance of the pharmacological properties of their byproducts. The functional properties of protein isolates from the byproducts of *Cucurbit moschata*, *Citrullus lanatus*, and *Lagenaria siceraria* have been well-investigated. A study to correlate the inhibition of free radicals with the concentrations of globulin hydrolysates prepared from the seeds of the *Cucurbitaceae* family showed that *Cucurbitaceae* seed-derived hydrolysates exhibited good antioxidant properties (51). The antioxidant activities of pumpkin (*Cucurbita pepo*) seed protein were reported in CCl_4_-induced liver injury (52). *Cucurbitaceae* seed protein hydrolysates showed higher antioxidant activity than unhydrolyzed seed protein, mainly due to the generation of bioactive peptides (53). The bioactivities are based on various factors, including the type of enzyme used for hydrolysis, the degree of hydrolysis, and the molecular weight distribution of the peptides. Furthermore, higher degrees of hydrolysis and smaller sizes of peptides are the key factors in the antioxidant effect. Although several *in vitro* studies have been performed, well-designed *in vivo* studies are still needed to confirm the antioxidant effects of *Cucurbitaceae* plant proteins on animals and humans.

### 4.2. Crude extracts that eliminate oxidative stress

Crude extracts that form during food processing are considered an alternative source of antioxidants for improved management and prevention of metabolic diseases associated with oxidative stress (54, 55). The major antioxidants found in plant extracts are phenolic compounds, which include phenolic acids, flavonoids, and anthocyanins (47). Numerous *in vitro* and *in vivo* studies have reported the effects of plant extract supplementation on oxidative stress in blood and peripheral tissues in humans (56). *In vitro* assays were performed on Canola and *Brassica napus* L. extracts using different extraction techniques to assess their antioxidant activities. Crude canola meal extract exhibited the highest antioxidant capacity in reducing H2O2-induced cytotoxicity, followed by sinapic acid, deodistillate extract, and accelerated canola meal solvent extract (45). *Clinacanthus nutans*, which belongs to the *Acanthaceae* family and is widely used as a traditional medicine, was examined and suppressed oxidative stress. Different phenolics were detected in these extracts, and protocatechuic acid was one of the most abundant. Aqueous methanolic leaf extract of *Clinacanthus nutans* was tested against hyperlipidemia-induced oxidative stress in rats. Leaf extracts attenuated oxidative stress by stimulating serum antioxidant enzyme activity and increasing hepatic antioxidant gene expression (46). Defatted *Canarium odontophyllum* pericarp and peel crude extracts reduced oxidative stress and lipid peroxidation *in vitro*. *Canarium odontophyllum* fruit is high in anthocyanins and possesses a stronger antioxidant capacity than most phenolic compounds. The results indicated that the crude extracts from the pericarp and peel have no toxic effects on human cell lines. However, extract derived from the peel of *Canarium odontophyllum* has a higher inhibitor effect against oxidative stress than the pericarp extract and could be used as a potential cardioprotective agent (47).

## 5. Plant-derived functional foods that regulate circadian rhythm

A number of studies have focused on circadian rhythm regulation by the crude extracts of natural source foods (Table 3). However, most studies have not examined the regulatory mechanisms of these crude extracts with respect to the circadian clock. The extract of tea could reverse daily circadian rhythm gene transcription (including Bmal1, Sirt1, Cry1/2, Per3, and Nampt) and relevant proteins in the liver and hypothalamus, leading to a series of physiological changes, including a decrease in blood pressure, the attenuation of insulin resistance and glucose/lipid metabolism regulation (57). After oral administration of proanthocyanidin obtained from grape seeds, the circadian genes Clock, Bmal1, Per2, Rorα, Rev-erbα, Nampt, and NAD were stimulated, which was accompanied by changes in melatonin and metabolite oscillation and lipid/glucose regulation in rats (58). *Hericium erinaceus* is an herbal medicine used in various Asian countries. The extract of *Hericium erinaceus* showed effects on behavioral rhythm, including the offset time of activity, sleep-wake cycle, and wakefulness around the end of the active phase (59). The extracts of *Polyporus* and *Bupleuri* radix induced phase delay and amplitude enhancement in mice and embryonic fibroblasts (MEFs) (60). Passionflower (*Passiflora incarnata Linnaeus*) held Generally Recognized as Safe (GRAS) status for use in foods in the USA and was listed in the European Pharmacopeia for having sedative effects. Passionflower extracts were reported to enhance dopamine and metabolic enzyme regulation by mediating Bmal1, Clock, Per1/2, and Cry1/2 (61). Treatment with momordin from bitter melon promoted PPARδ activity by upregulating PPARδ production and activation in HepG2 cells (62). The water extract of bitter melon (*Momordica charantia* L.) improved sleep quality and facilitated sleep by regulating the expression levels of the Clock, Per2 and Arntl genes (63).

**TABLE 3:** The effects of crude extracts from natural sources on circadian clock regulation.

Active extracts	Main source	Experimental model	Targets	Beneficial effects	References
Extract of tea	Tea	Male malignant stroke-prone spontaneously hypertensive rats	N/A	Decrease circadian rhythm of heart rate; Inhibit high blood pressure	(64)
		C57BL/6J mice	BMAL1, SIRT1, PER3, CRY1/2, NAMPT	Enhance amplitude; Attenuate insulin resistance; Regulate glucose/lipid metabolism; Prevent memory impairment.	(57)
		Human neuroblastoma SH-SY5Y cell	BAML1	Enhance amplitude; Protect against neural redox imbalance and mitochondrial dysfunction.	(65)
Proanthocyanidin	Grape seed	Male Wistar rats	BAML1, CLOCK, PER2, RORα, REV-ERBα, NAMPT, NAD	Regulate lipid/glucose metabolism Alter melatonin and metabolite oscillation	(66)
		HepG2 cells	BAML1, CLOCK, CRY.PER2, RORα, REV-ERBα	Shift the circadian phase	(58)
Extract of *Hericium erinaceus*	*Hericium erinaceus*	Male CBA/N mice	N/A	Affect offset time of activity Regulate sleep	(59)
Water solution of *Polyporus* and *Bupleuri radixa*	Polyporus and Bupleuri radix	Heterozygous PER2: LUC knock-in male mice MEFs	N/A	Delay circadian rhythm phase; Enhance amplitude	(60)
Extract of passionflower	*Passiflora incarnata Linnaeus*	NIH3T3 cells Male ICR mice	BAML1, CLOCK, PER1/2, CRY1/2	Induce high amplitude; Increase dopamine levels Affect synthetic and metabolic enzymes	(61)
Water extract of bitter melon	Bitter melon (*Momordica charantia* L.)	HepG2 cells	CLOCK, PER2, and ARNTL	Improve sleep quality Facilitate sleep	(63)

BMAL1, brain and muscle ARNT-Like 1; SIRT1, sirtuin 1; PER3, period circadian protein homolog 3 protein; CRY, pesticidal crystal proteins; NAMPT, nicotinamide phosphoribosyl transferase; CLOCK, circadian locomotor output cycles kaput; RORα, RAR-related orphan receptor alpha; NAD, nicotinamide adenine dinucleotide; ARNTL, aryl hydrocarbon receptor nuclear translocator-like protein 1.

## 6. Conclusion

An increasing number of plant-based functional foods have been reported to be effective in preventing metabolic diseases, some of which have been identified as plant proteins, peptides or essential amino acid residues. As circadian clock regulators, the rest of these factors have been tested as crude extracts without isolating the active components. Even for the identified plant protein, efficacy has been mostly tested *in vitro*. To date, no robust *in vivo* results have been reported, and convincing clinical studies are lacking. In addition to the lack of studies validating the mechanism of action and efficacy in humans, oral administration also limits the potential use of active plant protein-containing functional foods. The abundant microbiome in the gut may degrade these functional proteins or have undesirable effects. The relatively low absorption rate limits these active components prevent them from reaching the liver to regulate glucose and lipid mechanisms. We are sure that orally ingested plant proteins have the potential to regulate lipid and glucose metabolism and affect the expression levels of inflammation-, oxidative stress- and circadian clock-related genes. However, it is still unclear whether these signaling pathways are essential for bioactivity. In conclusion, there is still much to be explored in this area to validate plant-based functional foods as an effective approach to prevent metabolic diseases.

## Author contributions

S-hY and GT wrote the original manuscript. YS and J-qH conceived and designed the study. All authors participated in drafting and revising the manuscript before its final submission and approved the submitted version.
